# Disparities in pain management among patients with life-limiting illnesses in Latin America: a scoping review

**DOI:** 10.1186/s12904-026-02068-2

**Published:** 2026-03-20

**Authors:** Mariana González Garcés, Jerónimo Cárdenas Montoya, María Isabel Peña Martínez, Juanita Valencia García, Erwin Hernando Hernández Rincón

**Affiliations:** 1 Primary Care Physician, Universidad de La Sabana, Chía, Colombia; 2 Department of Family Medicine and Public Health, Universidad de La Sabana, University Campus Puente del Común, km 7 Autopista Norte, Chía, Colombia

**Keywords:** Pain management, Palliative care, Latin America, Health disparities, Opioids, Access to care, End of life care, Health equity

## Abstract

**Introduction:**

Patients with life limiting illnesses frequently experience moderate to severe pain, and many do not receive adequate analgesic control. In Latin America, palliative care coverage remains limited and access to essential opioids is often restricted, contributing to important inequities in pain management.

**Objective:**

To map and characterize the available evidence on disparities in pain management among patients receiving palliative care in Latin America, with particular attention to structural, cultural, and educational determinants associated with unequal access and treatment.

**Methods:**

A scoping review was conducted in three indexed databases, PubMed, Scopus, and Web of Science, and supplemented by snowball searching. Articles published between January 2019 and June 2025 were screened, yielding 511 records in total. After removal of duplicates and application of predefined inclusion and exclusion criteria, 19 studies were included in a thematic synthesis.

**Results:**

The evidence was organized into seven thematic categories: identified disparities; access to and availability of analgesic treatments; training of healthcare professionals; pain perception and patient and family satisfaction; clinical consequences associated with inadequate pain management; recommendations reported in the literature; and knowledge gaps and areas for future research. The findings suggest that disparities are associated with limited service distribution, regulatory and administrative barriers affecting opioid access, insufficient professional training, and sociocultural influences shaping pain perception and treatment decisions.

**Conclusion:**

The available evidence indicates that important inequities in pain management persist across Latin America. These disparities appear to be linked to structural constraints within health systems, uneven service distribution, regulatory challenges in opioid access, and gaps in professional education. Coordinated regional efforts, including strengthened training, improved access to essential medicines, and culturally responsive care strategies, may contribute to advancing more equitable palliative care delivery. Further context specific research is needed to inform policy development and implementation.

## Introduction

The International Palliative Care Association defines palliative care as the active and holistic care of individuals of all ages living with serious illnesses, particularly those in the terminal stage. Its primary goal is to improve the quality of life of patients, their families, and their caregivers [1]. The World Health Organization (WHO) also recognizes palliative care as a key component of universal health coverage [2].

Increasing life expectancy in recent years has been accompanied by a rise in chronic disease prevalence. In Latin America, 70% to 75% of deaths are attributable to these conditions, underscoring the urgent need to ensure adequate palliative care coverage for the entire population [2–4]. Currently, only 14% of individuals who require palliative care have access to it [2].

The WHO highlights Latin America as one of the regions with the greatest disparities in healthcare access, characterized by inequities in financing, the absence of national palliative care policies, limited opioid availability, inadequate integration of the specialty within health systems, and significant barriers in rural and low-income areas. These factors hinder palliative management and effective symptom control [5].

Consequently, many patients in the region die without adequate pain relief, despite the availability of analgesics and professional knowledge to provide proper management. Key barriers include fear of opioid addiction, lack of familiarity with institutional protocols, and inadequate follow-up due to geographical challenges. These issues are further exacerbated by limited coordination between primary care and palliative care, resulting in delayed symptom relief and prolonged suffering—particularly among those who are no longer candidates for curative treatment [5].

Although palliative care encompasses multiple dimensions, including psychological, social, and spiritual support, this review specifically focuses on pain management. Pain is the most prevalent and distressing symptom among patients with life-limiting illnesses, and its inadequate control represents both a clinical and ethical problem. Addressing this dimension in isolation is justified given its high impact on patient suffering, its central role in palliative care, and the structural inequities that restrict timely access to effective pain relief in Latin America.

This scoping review focuses on disparities in pain management among patients with life-limiting illnesses who receive—or are eligible to receive—palliative care services in Latin America. Throughout the manuscript, we use patient-centered terminology (e.g., “patients with life-limiting illness,” “patients nearing the end of life”) to emphasize the clinical context and avoid ambiguous uses of “terminal.”

## Methodology

A scoping review was conducted in June 2025, following a protocol registered in the Open Science Framework [6] and reported according to the Preferred Reporting Items for Systematic Reviews and Meta-Analyses extension for Scoping Reviews (PRISMA-ScR) [7]. The aim of this scoping review was to map and synthesize existing evidence on disparities in pain management among patients with life-limiting illnesses receiving palliative care in Latin America.

A scoping review design was selected because the topic of disparities in pain management within palliative care in Latin America is broad and heterogeneous, encompassing studies of various designs (qualitative, quantitative, mixed-methods, and reviews). This approach is particularly suitable when the available evidence is fragmented and unevenly distributed across countries. It allows the identification of key concepts, types of evidence, and gaps in knowledge—rather than assessing study quality or effect size.

### Search strategy

The search was performed in three electronic databases—PubMed, Scopus, and Web of Science—using the following research question: What are the main disparities in pain management in palliative care across Latin American countries, and what structural, clinical, or cultural factors influence their persistence?

Search terms corresponded to DeCS and MeSH headings such as pain, opioids, disparities, barriers, access to care, South America, and Central America. Grey literature was added through the snowball technique to capture additional relevant studies. The search included articles published between 2019 and 2025 in English or Spanish. Table 1 summarizes the detailed search strategy for each database.

**Table 1:** Search strategy across databases

Inclusion criteria	Exclusion criteria
Studies published between January 2019 and June 2025.	Publications before 2019.
Publications in Spanish or English.	Studies in other languages for which translations are not available.
Original articles, systematic reviews, qualitative, quantitative, or mixed studies.	Opinions, letters to the editor, editorials, and individual case reports.
Research conducted in Latin American countries.	Studies focused exclusively on other regions without analysis applicable to the Latin American context.
Articles that address clinical, structural, regulatory, or cultural aspects of pain management.	Theoretical studies without clinical application or empirical data.
Publications that include clinical experiences, barriers, or best practices in settings such as ICUs, emergency departments, or palliative care.	Studies not related to hospital or clinical care contexts.
Search algorithm	
PubMed (pain OR analgesia OR opioid OR “pain management” OR morphine) AND (“access to opioids” OR “opioid availability” OR disparities OR inequities OR barriers OR “regulatory restrictions” OR “access to care”) AND (“Latin America” OR “South America” OR “Central America” OR “Caribbean” OR Brazil OR Mexico OR Argentina OR Colombia OR Peru OR Chile OR Cuba OR “Puerto Rico”)	
Scopus (pain OR analgesia OR opioid OR “pain management” OR morphine) AND (“access to opioids” OR “opioid availability” OR disparities OR inequities OR barriers OR “regulatory restrictions” OR “access to care”) AND (“Latin America” OR “South America” OR “Central America” OR “Caribbean” OR Brazil OR Mexico OR Argentina OR Colombia OR Peru OR Chile OR Cuba OR “Puerto Rico”)	
Web of Science (pain OR analgesia OR opioid OR “pain management” OR morphine) AND (“access to opioids” OR “opioid availability” OR disparities OR inequities OR barriers OR “regulatory restrictions” OR “access to care”) AND (“Latin America” OR “South America” OR “Central America” OR “Caribbean” OR Brazil OR Mexico OR Argentina OR Colombia OR Peru OR Chile OR Cuba OR “Puerto Rico”)	

### Selection of studies

Two independent reviewers performed the initial screening based on predefined inclusion criteria. The initial search yielded 511 records across the three databases. After removing 153 duplicates using Rayyan software [8], 358 unique references remained for title and abstract screening. Of these, 28 articles met the eligibility criteria and were retained, while 11 additional papers were identified through snowball searching, for a total of 39 full-text articles assessed for eligibility. After applying the inclusion and exclusion criteria, 19 articles were included in the final qualitative synthesis.

### Inclusion and exclusion criteria

These criteria were defined according to the variables of population, concept, and context (PCC strategy). These include studies published between January 2019 and June 2025; publications in Spanish or English; original articles; systematic reviews; qualitative, quantitative, or mixed studies; research conducted in Latin American countries; articles that address clinical, structural, regulatory, or cultural aspects of pain management; and publications that include clinical experiences, barriers, or best practices in settings such as intensive care units, emergency departments, or palliative care. Studies focusing exclusively on other regions, theoretical papers without clinical data, opinions, letters to the editor, editorials, and single case reports were excluded.

In this review, study quality was not used as an exclusion criterion, since the objective was to map the available evidence rather than to grade its quality. However, the type of study, methodology, and main limitations were recorded to inform interpretation of the findings.

### Data extraction and tabulation

Data from the included studies were organized into a synthesis table (Table 2) covering key variables such as authors, year, country, study type, and thematic category. Categories included identified disparities; access and availability of analgesic treatments; training of health personnel; pain perception and patient/family satisfaction; clinical outcomes associated with inadequate pain management; study recommendations; and knowledge gaps and areas for future research.

**Table 2:** Matrix summary of included studies including objectives, design, context, main findings and key limitations

Authors (Year)	Country	Objective	Study design	Population / Unit of analysis	Setting	Key findings	Main limitations
León MX et al. (2022) [9]	Colombia	To assess availability and accessibility of opioids for pain and palliative care	Cross sectional survey study	Physicians prescribing opioids	Hospitals and primary care centers	Limited opioid availability and restrictive authorization processes, especially in rural areas	Self reported data; limited generalizability outside Colombia
Pastrana T et al. (2022) [10]	Latin America	To evaluate progress in palliative care development using macro indicators	Quantitative macro indicator analysis	National palliative care services	National health systems	35% of countries have fewer than one palliative care service per million inhabitants	Reliance on secondary national indicators; heterogeneity between countries
Sánchez Cárdenas MA et al. (2024) [11]	Colombia	To analyze geographic accessibility to palliative care services	Quantitative geographic analysis	Palliative care services	Rural and jungle regions	Severe geographic disparities with concentration of services in urban areas	Focused on one country; service availability does not measure quality
Ramos Guerrero JA et al. (2021) [12]	Mexico	To compare palliative care needs with installed capacity	Quantitative capacity analysis	Primary care units	Primary care and rural clinics	Absence of structured palliative care coverage in rural primary care settings	Institutional data; limited external validity
Bonilla P (2021) [13, 14]	Latin America	To synthesize regional development of palliative care	Systematic review	Regional literature	Latin American healthcare systems	Urban concentration of services and workforce shortages limiting rural access	Narrative heterogeneity; lack of standardized outcome measures
Pastrana T et al. (2021) [15]	Colombia	To examine universality of palliative care within the health system	Qualitative health policy analysis	Physicians and policy stakeholders	Health system and authorized pharmacies	Insurance authorization barriers and pharmacy related restrictions	Qualitative design; potential respondent bias
Lincoln SB et al. (2019) [16]	Mexico	To describe cancer pain management and opioid regulation	Narrative review	Published studies on cancer pain	Oncology and palliative services	Regulatory restrictions contribute to low opioid availability, particularly oral morphine	Narrative methodology without formal quality appraisal
Garzón Duque MO et al. (2023) [17]	Latin America	To discuss opioid use in chronic and terminal pain management	Narrative review	Medical education and prescribing practices	Academic and clinical contexts	Limited formal opioid training in medical curricula	Non systematic review; no empirical data
Judkins J et al. (2021) [18]	Bolivia	To conduct a national situational analysis of palliative care	Quantitative situational analysis	Health institutions	National health system	Lack of accredited national palliative care training programs	Descriptive data; limited outcome assessment
Goodman Meza D et al. (2021) [19]	Mexico	To analyze geographic and socioeconomic disparities in opioid access	Retrospective secondary data analysis	Patients receiving opioids	National surveillance database	Significant geographic and socioeconomic disparities; fear of opioid use limits acceptance	Surveillance database limitations; no qualitative exploration
Pergolizzi J et al. (2023) [20]	Latin America	To review pain assessment perspectives in cancer patients	Narrative review	Regional cancer pain literature	Multi country perspective	Cultural and spiritual factors influence pain perception and opioid acceptance	Narrative design; lack of comparative data
Vahos J et al. (2023) [21, 22]	Multi country Latin America	To explore perceived barriers to opioid access among professionals	Quantitative observational study	Health professionals	Palliative care services	Fear of adverse effects and distrust toward opioids among providers	Self reported perceptions; cross sectional design
Ferreira S et al. (2019) [23]	Brazil	To assess oncology nurses knowledge of cancer pain management	Cross sectional study	Oncology nurses	Tertiary cancer center	Knowledge gaps associated with inadequate pain control practices	Single center study; limited generalizability
Vargas Escobar LM et al. (2022) [14]	Colombia	To map barriers to palliative care access using stakeholder input	Qualitative social mapping study	Patients and healthcare providers	Hospitals	Fragmented care pathways and long waiting times increase suffering and costs	Qualitative context specific findings
Pérez Cruz PE et al. (2023) [22]	Chile	To evaluate serious health related suffering and palliative coverage	Mixed method health systems analysis	National population	National health system	Partial coverage contributes to avoidable suffering and increased emergency utilization	Country specific analysis; modeling assumptions
Luna Meza A et al. (2021) [24]	Colombia	To explore decision making in end of life cancer care	Qualitative phenomenological study	Clinicians	Oncology and palliative services	Communication and decision making barriers affect symptom control	Small qualitative sample
Pergolizzi J et al. (2024) [25]	Latin America	To describe challenges in palliative care development	Narrative review	Regional literature	Multi country analysis	Structural policy gaps and limited telemedicine implementation	Narrative synthesis without methodological appraisal
Sánchez Cárdenas MA et al. (2021) [26]	Colombia	To assess development of palliative care using international indicators	Quantitative observational study	Palliative care services	National health system	Persistent geographic inequalities in service distribution	Country level ecological analysis
Sánchez Cárdenas MA et al. (2023) [27]	Colombia	To develop an action plan to reduce palliative care inequality	Mixed method implementation study	Palliative care programs	Hospitals	Multistakeholder strategies and digital tools proposed to improve coordination	Early implementation phase; limited outcome evaluation

Two reviewers independently extracted the data. Discrepancies were resolved through discussion, and a third reviewer intervened when consensus could not be reached. Agreement between reviewers was monitored, and conflicts were resolved systematically to ensure consistency, traceability, and reliability of the data.

### Data analysis and synthesis

We conducted an inductive thematic synthesis following Braun and Clarke’s framework for thematic analysis (familiarization, initial coding, theme development, review, definition, and reporting). Two reviewers independently coded the extracted findings and iteratively grouped codes into higher-order categories. Discrepancies were resolved by discussion until consensus was achieved; a third reviewer adjudicated unresolved issues. An audit trail documented coding rules, category evolution, and decision rationale to enhance transparency and reproducibility. Although kappa statistics were not calculated, double coding and consensus procedures ensured internal consistency throughout the analysis.

## Results

**Fig. 1:**
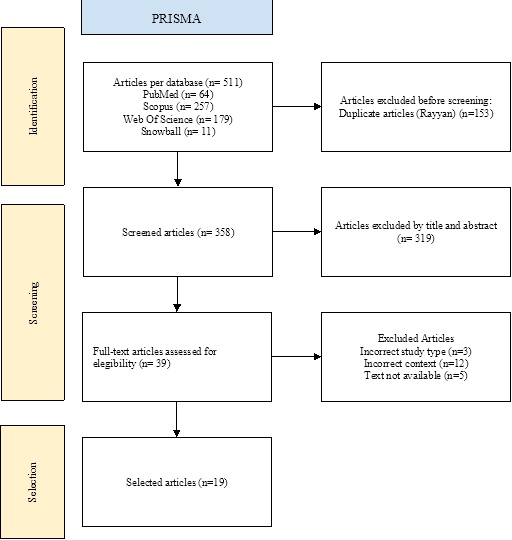
PRISMA flow diagram showing article selection process

A total of 511 articles were identified across the three databases using the predefined search strategy and snowball methodology. After applying the inclusion criteria (study type, publication year, language, and regional context), 358 non-duplicate records remained. Following title and abstract screening, 319 were excluded, leaving 39 full-text papers reviewed for eligibility. Finally, 19 studies met all inclusion criteria and were retained for qualitative synthesis (Fig. 1).

The most common study design was quantitative observational (*n* = 7; 36.8%), followed by reviews (*n* = 6; 31.6%), qualitative studies (*n* = 3; 15.8%), mixed-methods designs (*n* = 2; 10.5%), and one retrospective study (*n* = 1; 5.3%).

Geographically, most studies were conducted in Colombia (*n* = 12; 63.2%), Mexico (*n* = 7; 36.8%), Chile (*n* = 5; 26.3%), Argentina (*n* = 4; 21.1%), Brazil and Peru (*n* = 3; 15.8%), and Bolivia (*n* = 2; 10.5%). The most frequent publication years were 2021 (*n* = 6; 31.6%), 2023 (*n* = 5; 26.3%), 2022 (*n* = 4; 21.1%), and 2019–2024 (*n* = 2; 10.5%).

**Fig. 2:**
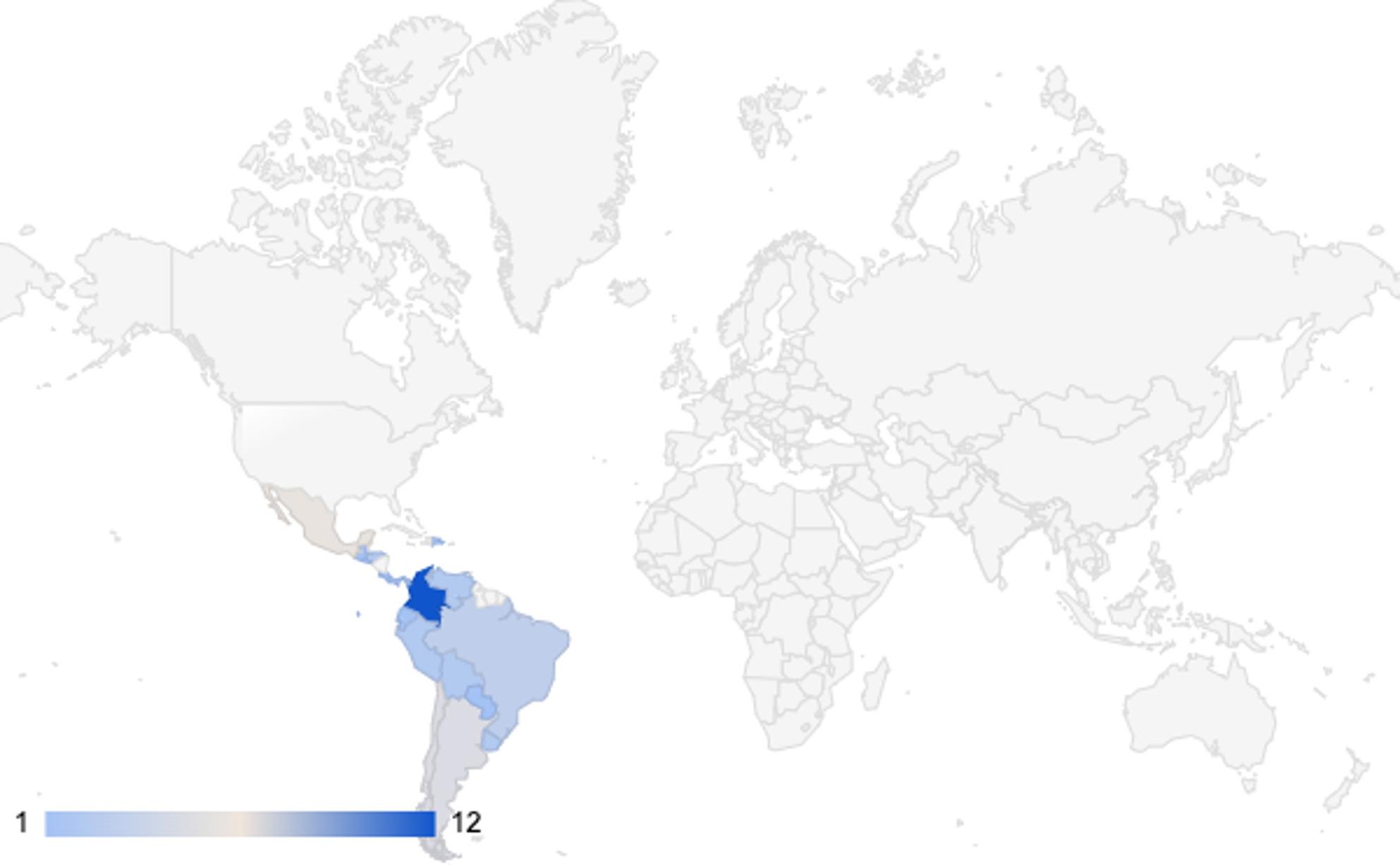
Geographic distribution of the included articles. (A single article may be represented in multiple countries)

Seven thematic categories were identified: (a) disparities in pain management; (b) access to and availability of analgesic treatments; (c) training of healthcare personnel; (d) pain perception and patient/family satisfaction; (e) clinical outcomes associated with inadequate pain management; (f) recommendations from included studies; and (g) knowledge gaps and areas for future research. Table 3 summarizes these categories and their corresponding studies. Figures and tables illustrate the distribution of publications by country and theme (Fig. 2).

**Table 3:** Thematic categories and number of included studies

Thematic category	Number of studies	Percentage
Disparities in pain management	4	21.1%
Access and availability of analgesic treatments	3	15.8%
Training of health personnel	2	10.5%
Pain perception and patient/family satisfaction	3	15.8%
Clinical outcomes of inadequate pain management	3	15.8%
Study recommendations	2	10.5%
Knowledge gaps/areas of future research	2	10.5%
Total	19	100%

## Discussion

Palliative care has been shown to alleviate suffering, control symptoms, and improve quality of life for patients, their families, and caregivers [28]. In Latin America, however, important disparities persist in both access to and quality of pain management. The available evidence suggests that these inequities are multifactorial, involving geographic, structural, cultural, and educational determinants that affect all levels of care.

### Structural and geographic disparities

Several studies highlight marked inequalities in access to pain management for patients with life limiting illnesses across Latin America. Approximately 35% of countries report fewer than one palliative care service per million inhabitants [9, 10]. This shortage limits coverage, particularly in rural and peripheral regions, where long travel times and inadequate transportation hinder timely access [11]. In Mexico, for instance, none of the 1,003 primary care units in rural areas have palliative care teams [12]. Workforce shortages, limited professional recognition, and insufficient incentives appear to contribute to service fragmentation and restricted outreach to underserved populations [13].

### Access to essential opioids

Access to opioids remains uneven, largely associated with bureaucratic authorization processes, inconsistent procurement systems, and disparities between contributory and subsidized insurance regimes [9, 15, 16]. In Colombia, 76% of surveyed physicians reported that first level facilities lack both palliative care services and opioid availability. Similar challenges are described across the region, where limited pharmaceutical infrastructure, supply shortages, and regulatory restrictions further exacerbate access gaps.

Several studies indicate that patients managed within structured palliative care programs may experience improved access to essential opioids, facilitated by streamlined prescription protocols and the presence of trained personnel [15, 16, 29]. However, this potential benefit appears to be unevenly distributed across countries and health systems in Latin America.

### Professional training deficits

Across the region, fewer than 15% of medical schools include formal palliative care education, and postgraduate training opportunities remain limited [17, 30]. In Bolivia, for example, no accredited training programs currently exist [18]. These gaps are associated with insufficient familiarity with clinical guidelines, misconceptions regarding opioid use, and concerns about addiction, which may contribute to underprescription and inadequate pain control. Expanding academic training and professional development in palliative care may represent an important component in broader efforts to improve equitable pain management.

### Cultural and spiritual influences

Cultural, social, and spiritual factors strongly shape pain perception and expression. Concepts such as resigned endurance, religious interpretations of suffering, and fear of addiction influence care seeking behaviors [19, 20]. These observations are consistent with findings by Dittborn et al. [31], who reported that cultural interpretations of suffering and family communication barriers significantly affect acceptance of palliative care and decision making around end of life pain management in Latin America.

Low health literacy and reliance on traditional remedies, often perceived as safer or more accessible, may further delay appropriate treatment [32]. As noted by Vahos et al. [21], these dynamics can also foster mistrust between patients, families, and healthcare providers, underscoring the importance of culturally responsive communication strategies.

### Clinical consequences

Inadequate pain management negatively affects patient wellbeing. It may undermine trust in healthcare teams, reduce adherence to treatment, and diminish quality of life [14, 23]. Long waiting times and systemic fragmentation, particularly in Colombia and Chile, are associated with delays in care and increased financial and emotional burden [14, 22]. These findings reinforce the ethical importance of promoting equitable access to pain relief as a component of high quality end of life care.

### Knowledge gaps and future directions

The existing literature reveals substantial gaps, including limited availability of validated pain assessment tools, particularly in oncology and palliative contexts, and insufficient research addressing spiritual pain and rural populations (26 to 28). Future research may benefit from interdisciplinary approaches and the development of culturally appropriate assessment instruments that reflect the sociocultural and systemic realities of Latin American countries.

### Limitations of the current evidence base

Despite the relevance of the findings, several limitations within the current evidence base should be acknowledged. First, a substantial proportion of the included studies originate from Colombia and Mexico, introducing a potential regional concentration that may not accurately represent the heterogeneity of all Latin American countries. Health systems, regulatory frameworks, opioid policies, and palliative care development vary considerably across the region, limiting the generalizability of findings.

Second, a notable proportion of the included literature consists of narrative reviews and descriptive analyses. While these contributions are valuable for contextual understanding, they may limit the overall strength of inference due to methodological heterogeneity and the absence of standardized outcome measures.

Third, no formal methodological quality appraisal was conducted, consistent with the objectives of a scoping review following PRISMA ScR guidelines. However, this approach precludes formal assessment of risk of bias across studies.

Finally, publication bias cannot be excluded, as most available studies originate from countries with more established academic and research infrastructures in palliative care. As a result, contexts with limited research capacity may remain underrepresented in the literature.

These considerations highlight the need for more robust, multicountry empirical research to strengthen the regional evidence base.

### Path forward

Addressing disparities in palliative pain management will likely require coordinated strategies, including strengthened regulatory frameworks, improved opioid availability, expanded palliative care education at all levels of training, and the integration of culturally responsive care models. Such approaches may contribute to more equitable and person centered end of life care across the region.

Efforts to reduce inequities may also involve broader health system reforms. National palliative care plans could incorporate equitable opioid distribution mechanisms, structured professional training pathways, and the inclusion of palliative care indicators within universal health coverage frameworks. Regional collaboration through Latin American observatories and partnerships with international organizations such as the World Health Organization may support the development of consistent standards and evidence informed policies aimed at improving equity in end of life pain management [33].

## Conclusions

The available evidence suggests that important disparities in pain management across Latin America appear to be associated with limited opioid availability, insufficient professional training, and the absence of consistent public policies for palliative care. The available evidence also highlights the influence of cultural beliefs, communication barriers, and the lack of standardized assessment tools, contributing to fragmented care and compromised quality of life for patients with life-limiting illnesses and their families.

Addressing these disparities will likely require coordinated regional efforts. Strengthening palliative care education, promoting equitable access to essential opioids, and integrating culturally sensitive approaches into national health strategies may represent important steps toward improving the quality and equity of end-of-life care across Latin America. Further context-specific research is needed to guide policy development and implementation.

## Data Availability

All data generated or analyzed during this study are included in this published article and its supplementary information files.
